# Exposure to Low Doses of Biocides Increases Resistance to Other Biocides and to Antibiotics in Strains of *Listeria monocytogenes*

**DOI:** 10.3390/biology14050495

**Published:** 2025-05-01

**Authors:** Cristina Rodríguez-Melcón, Rosa Capita, Carlos Alonso-Calleja

**Affiliations:** 1 Department of Food Hygiene and Technology, Veterinary Faculty, University of León, E-24071 León, Spain; 2 Institute of Food Science and Technology, University of León, E-24071 León, Spain

**Keywords:** *Listeria monocytogenes*, disinfectants, low doses, adaptation, antibiotic resistance

## Abstract

The effect of sub-inhibitory doses of three disinfectants, sodium hypochlorite (SHY), peracetic acid (PAA), and benzalkonium chloride (BZK), on the resistance to biocides and antibiotics of five strains of *Listeria monocytogenes* was determined. The minimum inhibitory concentration (MIC) of these chemicals ranged (ppm) between 3533.3 ± 28.9 and 3783.3 ± 28.9 for SHY, between 1000.0 ± 25.0 and 1050.0 ± 25.0 for PAA, and between 1.3 ± 0.6 and 4.3 ± 0.6 for BZK. The minimum bactericidal concentration (ppm) was between 3683.3 ± 57.7 and 3983.3 ± 28.9 for SHY, between 1050.0 ± 25.0 and 1250.0 ± 25.0 for PAA, and between 1.7 ± 1.2 and 4.7 ± 1.2 for BZK. Exposure of the strains to increasing sub-inhibitory concentrations of the biocides caused adaptation and cross-adaptation to these substances, markedly so in the case of BZK, relative to which some strains saw their MIC value increase up to 5.2 times after being exposed to low doses of this disinfectant. After exposure to biocides, changes in the resistance to antibiotics of the strains were also observed. In some cases, strains moved from a category of susceptible or of reduced susceptibility to resistant, especially when exposure was to SHY. These findings suggest a need to avoid the application of sublethal concentrations of disinfectants in both the food industry and the healthcare system.

## Simple Summary

Listeriosis is a serious foodborne illness caused by the bacterium *Listeria monocytogenes*. In some cases, antibiotics are needed to treat this infection. Antibiotic-resistant bacteria are a public health challenge, as some antibiotics are invalidated as a therapeutic option. For this reason, it is important to avoid situations that cause an increase in antibiotic resistance of *L. monocytogenes*. In this research work, it was observed that contact with low doses of disinfectants widely used both in the food industry and in the healthcare system can increase the resistance of this microorganism to some antibiotics and disinfectants. Therefore, the use of disinfectants at concentrations below the lethal dose should be avoided.

## 1. Introduction

*Listeria monocytogenes* is a Gram-positive, facultative anaerobic, non-spore-forming bacterium responsible for listeriosis, a zoonosis whose main transmission route is through the consumption of contaminated food [1,2]. A total of 13 serotypes of *L. monocytogenes* have been described, denoted 1/2a, 1/2b, 1/2c, 3a, 3b, 3c, 4a, 4ab, 4b, 4c, 4d, 4e, and 7, although three of them (1/2a, 1/2b, and 4b) are responsible for the vast majority of human infections [3,4]. Serotype 4b strains are the etiological agent in most cases and outbreaks of listeriosis, while most strains isolated from foodstuffs and food-processing installations belong to serotype 1/2a [5].

In 2023, 2952 confirmed cases of invasive human listeriosis were reported in the European Union, amounting to 0.66 cases per 100,000 people. The mortality rate and the hospitalization rate were 19.7% and 96.5%, respectively, the highest values among all foodborne illnesses [6]. The elderly, pregnant women, neonates, and immunosuppressed adults are the groups suffering most from this disease in terms of both frequency and severity, although people not affected by these risk factors can also become infected. The most common clinical presentations of invasive listeriosis are sepsis, meningitis, encephalitis, spontaneous abortion, and stillbirth [7].

*L. monocytogenes* is a ubiquitous bacterium that can grow in various adverse environmental conditions. These include low temperatures, some strains being capable of multiplying at temperatures barely above 0 °C; a wide range of pH values, anywhere from 4.0 to 9.6; high salt content, as much as 10% to 20%; and low levels of oxygen. These characteristics constitute a major concern for food industries [8]. Contamination of food products by this pathogen can frequently occur during food processing, generally through cross-contamination events involving contact surfaces where it is present [9]. An additional cause for concern is the fact that this microorganism can persist for years in the equipment and installations of the food-processing industry, principally because of its capacity to survive and multiply in conditions that are stressful for other bacteria, as well as its ability to form biofilms [1,10].

Biofilms are heterogeneous communities of microbial cells embedded in a self-produced extracellular polymeric matrix that can present complex three-dimensional structures and adhere to inert or living surfaces [11,12]. The formation of biofilms by pathogenic bacteria is a concern for the food industry and in clinical environments since these structures represent a sizeable source of contamination. Furthermore, bacterial biofilms are more resistant to environmental challenges, including disinfectants and antibacterial agents, than planktonic or free-living cells, a situation that increases the survival rates of sessile bacteria [13].

Among the biocides most often used for cleaning and disinfection in food industries are sodium hypochlorite (SHY), peracetic acid (PAA), and benzalkonium chloride (BZK). SHY is a chlorine-based disinfectant that acts as an oxidizing compound, interacting with cell membranes or directly penetrating the cell, forming N-chlorine groups that react with the cellular metabolism. It is a substance that is widely employed because of its broad-spectrum bactericidal activity, powerful antimicrobial capacity, and low cost [10,14]. PAA, one of the most widely used antimicrobial compounds in dairy-based industries [15], has a broad spectrum of antimicrobial activity and is active at temperatures between 0 °C and 25 °C. Quaternary ammonium compounds (QAC) are unquestionably one of the most used groups of disinfectants in the food industry, being effective against different microbial groups, even at low concentrations [10]. Some compounds derived from quaternary ammonium, such as BZK, are cationic surfactants included mainly in disinfectant and antiseptic formulations used in healthcare facilities, agriculture, and industry [16]. SHY and PAA are approved for use in both the European Economic Area (EEA) and Switzerland for various uses, including disinfection in food-processing plants [17].

An increase in tolerance to biocides would pose a crucial public health problem, as it could be expected to contribute to greater persistence of pathogenic and spoilage bacteria in the food chain [18]. Inappropriate use of these substances, for example, their incorrect concentration or inappropriate storage, or the presence of excessive residues of organic matter, which reduce the effectiveness of some disinfectants, such as chlorinated compounds, may involve the exposure of bacteria to sublethal doses of the biocides, and this entails a risk that microorganisms may adapt to the compounds [19]. Furthermore, recent studies have shown that exposure to sublethal concentrations of biocides stimulates the production of biofilms by *L. monocytogenes* [12] and by other pathogenic microorganisms [17,18,19].

An additional phenomenon observed in recent years is growing resistance to antibiotics, a global threat to public health that involves both the main pathogenic microorganisms and the whole range of antimicrobial compounds [20]. Antibiotic resistance is acquired because of sporadic mutations or by the horizontal transfer of resistance genes, some of which may come from commensal microorganisms found in food [21]. An association has been demonstrated between contact with biocides and the emergence of antibiotic resistance. It has been hypothesized that some biocides and antibiotics may share target sites and consequently the resistance mechanisms developed by bacteria [22]. Hence, recent scientific evidence suggests that the selective pressure exerted by the use of biocides in sublethal concentrations could contribute to the expression and dissemination of antibiotic resistance mechanisms [18].

The objective of this research work was to determine whether exposure of several *L. monocytogenes* strains to sublethal doses of three disinfectants commonly used in food industries (SHY, PAA, and BZK) caused adaptation and cross-adaptation to biocides and influenced the susceptibility of strains to antibiotics. The differences between strains were explored.

## 2. Materials and Methods

### 2.1. Bacterial Strains and Culture Conditions

Five strains of *L. monocytogenes* available in our laboratory were used. These were ATCC 19114, ATCC 13932, and ATCC 15313 from the American Type Culture Collection; CETC 936 from the Spanish Collection of Type Cultures; and NCTC 11994 from the National Collection of Type Cultures. ATCC 19114 belongs to serotype 4a, ATCC 13932 to 4b, ATCC 15313 to 1/2a, CETC 936 to 1/2b, and NCTC 11884 to 4b. Bacterial cultures were stored at −80 °C in tryptone soy broth (TSB, Oxoid Ltd., Hampshire, UK) with 20% (*v*/*v*) glycerol. Before each experiment, cells were activated by transferring approximately 20 μL of the frozen culture to tubes with 5 mL of TSB (Oxoid) and subsequent incubation of the broth overnight at 37 °C. Cultures were plated on tryptone soy agar (TSA, Oxoid) and maintained at 4 °C ± 1 °C for later use.

### 2.2. Biocides

Three chemicals were used: sodium hypochlorite (NaClO, SHY, Sigma-Aldrich Co., St. Louis, MO, USA), benzalkonium chloride (BZK, Sigma-Aldrich), and peracetic acid (PAA, Sigma-Aldrich). For the preparation of the corresponding solutions, the concentration values for the substances used were 10% (expressed as a percentage of free chlorine) for SHY, 95% (assuming that the product was free from any impurity) in the case of BZK, and 39% for PAA. Solutions of each of the chemicals were prepared under aseptic conditions in sterile distilled water before the start of each experiment.

### 2.3. Calculation of Minimum Inhibitory Concentration (MIC)

Values for minimum inhibitory concentration (MIC) were established using the broth microdilution method described by the United States Clinical and Laboratory Standards Institute (CLSI) [23] with some modifications. Five colonies of each strain were taken from the TSA plates (Oxoid), inoculated into tubes with 9 mL of TSB (Oxoid), and incubated at 37 °C for 24 h to obtain a final concentration in each tube of approximately 5 × 10^8^ cfu/mL. Polystyrene microtiter plates with one hundred wells (Oy Growth Curves Ab Ltd., Helsinki, Finland) were used for the experiment. The wells were filled with 20 μL of the disinfectant solution: SHY, BZK, or PAA (each of these biocides in their range of concentrations indicated below in this section) and 180 μL of inoculum appropriately diluted in order to obtain a final concentration in each well of 5 × 10^5^ cfu/mL. The concentration of the inoculum was confirmed by plating. The range of concentrations of biocides used varied according to the compound. Initially wide ranges of biocides were used, and then the concentrations were delimited. The final concentrations tested for each compound in each strain differed by 50 ppm (SHY), 25 ppm (PAA), or 1 ppm (BZK). Positive and negative controls were also established. Growth was determined by measuring optical density (OD_420–580_) on a Bioscreen C MBR machine (Oy Growth Curves Ab Ltd.). The MIC was established as the minimum biocide concentration necessary to prevent bacterial growth after 48 h of incubation.

### 2.4. Calculation of the Minimum Bactericidal Concentration (MBC)

Minimum bactericidal concentration (MBC) values were established using the broth microdilution method in accordance with the CLSI [23], with minor modifications. From the microtiter plates (Oy Growth Curves Ab Ltd.) on which the corresponding MICs were determined after 48 h of incubation at 37 °C, 100 μL from the wells in which cessation of growth of the microorganism had been detected were streaked onto the surface of TSA plates (Oxoid). These plates were incubated for 48 h at 37 °C, MBC being established as the lowest concentration of biocide with which colonies were not formed under these conditions. Considering the fact that the detection limit of the surface seeding technique is 10 cfu/mL, an absence of growth on the TSA plates indicated that the concentration was lower than this value and, therefore, that the initial concentration (10^5^ cfu/mL) had been reduced to under 10 cfu/mL. Hence, MBC was defined as the minimum amount of biocide capable of destroying more than 99.99% of the bacteria present.

### 2.5. Adaptation of L. monocytogenes Strains to Biocides

The experiment was carried out in the same way as described for MIC determinations [17]. Five colonies of each strain were taken from TSA plates (Oxoid), then inoculated into tubes with 9 mL of TSB (Oxoid) and incubated at 37 °C for 24 h to obtain a final concentration of approximately 5 × 10^8^ cfu/mL. A total of 200 µL, comprising a mixture of inoculum at the appropriate dilutions (180 µL) and biocide (20 µL), was incorporated. Thus, the bacterial concentration was 5 × 10^5^ cfu/mL, whilst the concentration of biocides was set at the lowest MIC/2 values for each relative to the strains under study. When growth was observed, 20 µL of the suspension was transferred to the next well under aseptic conditions. This well contained 160 µL of TSB and 20 µL of the disinfectant solution at levels such that the final concentration in the well was 1.5 times that of the previous well. The procedure was repeated until growth was no longer observed after 72 h of incubation at 37 °C. The suspension from the last well where growth was noted was streaked onto TSA plates with the relevant biocide; half of the maximum concentration of biocide that allowed microbial growth was added to the TSA. After incubation at 37 °C for 48 h, the agar plates were stored at 4 °C ± 1 °C for up to one week, as necessary.

To carry out subsequent experiments, biocides at concentrations of MIC/2 were added to tubes with 9 mL of TSB, thus promoting the growth of adapted bacterial cells, while TSB without biocides was used for bacterial cells not previously exposed. The broth was inoculated with the strains preserved on TSA plates (Oxoid) with biocides in the case of cells adapted to SHY, BZK, or PAA or plain TSA plates (Oxoid) for cells that had not undergone adaptation.

### 2.6. Calculation of the MIC of Biocides for Strains Adopted to Them

As in the case of non-adapted strains, minimum inhibitory concentration (MIC) values were established using the broth microdilution method recommended by the CLSI [23]. Five colonies of each strain were taken from TSA plates (Oxoid) and inoculated into tubes with 9 mL of TSB (Oxoid) containing a biocide concentration of MIC/2, being incubated thereafter at 37 °C for 24 h. Polystyrene microtiter plates with one hundred wells (Oy Growth Curves Ab Ltd.) were used for the experiment. The wells were filled with a total of 200 μL, which comprised 20 μL of the relevant disinfectant solution (a range of concentrations being used) and 180 μL of inoculum at the appropriate dilution needed to obtain a final concentration in the well of approximately 5 × 10^5^ cfu/mL. In addition, positive and negative controls were established, the first incorporating 200 μL of the last dilution of the strain and the second 200 μL of sterile TSB. The inoculum concentration was confirmed by plating. Growth was determined by measuring OD_580_ in a Bioscreen C MBR (Oy Growth Curves Ab Ltd.). The MIC was deemed to be the minimum biocide concentration necessary to prevent bacterial growth after 48 h of incubation.

### 2.7. Calculation of the MIC of the Biocides for Strains Adapted to Them

Minimum bactericidal concentration (MBC) values were determined using the broth microdilution method described by the CLSI [23], with some modifications. From the microtiter plates (Oy Growth Curves Ab Ltd.) in which the corresponding MICs were determined after 48 h of incubation at 37 °C, 100 μL were taken from the wells of each adapted strain in which cessation of growth of the microorganism was detected, and they were plated on the surface of TSA plates (Oxoid) that were incubated for a further 48 h at 37 °C. The MBC was deemed to be the minimum amount of biocide used, capable of destroying more than 99.99% of the bacteria present.

### 2.8. Antibiotic Susceptibility Testing

Before and after exposure to disinfectants, the susceptibility of *L. monocytogenes* strains to thirty antibiotics of clinical interest was determined using the disc diffusion method [24]. The first step was to inoculate the strains into tubes with 9 mL of TSB (Oxoid) plus biocide at half of the MIC in the case of the adapted strains or in tubes with 9 mL of TSB without biocides in the case of those not-so-exposed, these being incubated for six hours at 37 °C. Thereafter, the entire surfaces of TSA plates were inoculated with the aid of a swab previously dipped into the bacterial suspension present in the TSB tubes. After this, the antibiotic discs, five per plate were deposited onto the plates, using sterile forceps. The following 30 different discs (Oxoid) were used: streptomycin (S, 10 µg), gentamycin (CN, 10 µg), amikacin (AK, 30 µg), kanamycin (K, 30 µg), vancomycin (VA, 30 µg), ampicillin (AMP, 10 µg), penicillin (P, 10 units), oxacillin (OX, 1 µg), amoxicillin (AML, 10 µg), ampicillin plus sulbactam (SAM, 20 µg), enrofloxacin (ENR, 5 µg), ciprofloxacin (CIP, 5 µg), moxifloxacin (MXF, 5 µg), nalidixic acid (NA, 30 µg), tetracycline (TE, 30 µg), clindamycin (DA, 2 µg), erythromycin (E, 15 µg), clarithromycin (CLR, 15 µg), imipenem (IMP, 10 µg), meropenem (MEM, 10 µg), cephalothin (KF, 30 µg), cefoxitin (FOX, 30 µg), cefotaxime (CTX, 30 µg), ceftriaxone (CRO, 30 µg), cefepime (FEP, 30 µg), sulfamethoxazole-trimethoprim (SXT, 25 µg), chloramphenicol (C, 30 µg), rifampin (RD, 5 µg), nitrofurantoin (F, 300 µg) and aztreonam (ATM, 30 µg). The plates were incubated for 24 h at 37 °C in an inverted position, whereafter the inhibition zones were measured, and the strains were classified as susceptible, intermediate (with reduced sensitivity), or resistant, according to guidelines published by the European Committee on Antibiotic Susceptibility Testing (EUCAST) [25] and by the CLSI [26]. A total of 600 tests were carried out, providing for all combinations of the five strains, the three biocides and the unexposed controls, and the 30 antibiotics.

### 2.9. Statistical Analysis

Three replications were performed on different days for each condition. Data were compared using nonparametric (Mann–Whitney U) tests. All analyses were carried out using the Statistica^®^ 8.0 package (StatSoft Ltd., Tulsa, OK, USA). Significant differences were established for a probability level of 5% (*p* < 0.05).

## 3. Results

### 3.1. Minimum Inhibitory Concentration (MIC) and Minimum Bactericidal Concentration (MBC) Values of Each Biocide for L. monocytogenes Strains

The MIC and MBC values are presented in Table 1. Likewise, the maximum concentrations of biocides that allowed microbial growth after several successive passages in a culture medium with gradually increasing concentrations of the compounds are shown, demonstrating adaptation.

Sodium hypochlorite (SHY) was the antimicrobial substance that required the highest concentrations to inhibit the growth of *L. monocytogenes* strains after 48 h of exposure, with MIC lying between 3533.3 ± 28.9 ppm and 3783.3 ± 28.9 ppm, equating to 353 ppm to 378 ppm of free chlorine. This was followed by peracetic acid (PAA), with an MIC of between 1000.0 ± 25.0 ppm and 1050.0 ± 25.0 ppm. The lowest MIC values were noted for benzalkonium chloride (BZK), with values between 1.3 ± 0.6 ppm and 4.3 ± 0.6 ppm. Regarding MBC values, figures equal to or greater than the MIC were found in all cases, ranging between 3683.3 ± 57.7 ppm and 3983.3 ± 28.9 ppm for SHY, between 1050.0 ± 25.0 ppm and 1250.0 ± 25.0 ppm for PAA, and between 1.7 ± 1.2 ppm and 4.7 ± 1.2 ppm for the BZK. As was true for MICs, SHY was the antimicrobial substance that required the highest concentrations to inactivate microorganisms.

After successive passages in a culture medium with increasing concentrations of biocides, the maximum concentration of SHY that allowed bacterial growth ranged between 2770.3 ± 1100.2 ppm and 6890.7 ± 1705.0 ppm, depending on the strain. For PAA, adaptation values of between 875.0 ± 216.5 ppm and 1312.7 ± 325.0 ppm were obtained. In the case of BZK, great differences were observed in the adaptation values of the different strains, as may be seen in Table 1 (from 2.0 ± 0.5 ppm to 9.9 ± 2.5 ppm).

**Figure 1:**
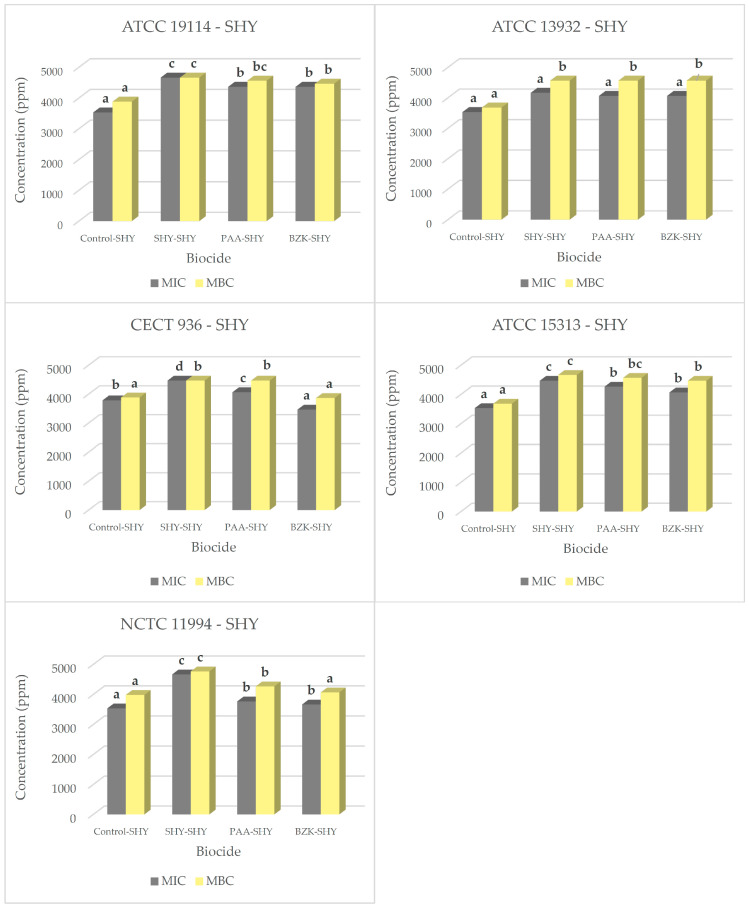
MICs and MBCs for sodium hypochlorite (SHY) in several *L. monocytogenes* strains. These were either not adapted (control) or previously adapted to sodium hypochlorite (SHY), peracetic acid (PAA), or benzalkonium chloride (BZK). MIC and MBC values for sodium hypochlorite (SHY) in strains not adapted to biocides (control–SHY), in strains adapted to sodium hypochlorite (SHY–SHY), in strains adapted to peracetic acid (PAA–SHY), and in strains adapted to benzalkonium chloride (BZK–SHY); *L. monocytogenes* ATCC 19114 serotype 4a; *L. monocytogenes* ATCC 13932 serotype 4b; *L. monocytogenes* CECT 936 serotype 1/2b; *L. monocytogenes* ATCC 15313 serotype 1/2a; *L. monocytogenes* NCTC 11994 serotype 4b. Data are the mean ± STD of three determinations. Data in the same graphics with no letters in common (superscript) are significantly different (*p* < 0.05).

Once the adaptation of the strains to the biocides had been achieved, the MICs and MBCs for the three biocides studied were recalculated. Table 2 shows the results for MICs and MBCs of SHY in the strains adapted to biocides. In most cases, the adapted strains showed an increase relative to the control strains in the MIC of SHY (Figure 1). MIC values for adapted strains ranged between 3466.7 ± 115.5 ppm and 4666.7 ± 57.7 ppm.

In respect of the MBCs, the strains adapted to SHY, PAA, or BZK in most cases presented higher values in comparison with the control strains. Values ranged between 4466.7 ± 115.5 ppm and 4766.7 ± 115.5 ppm for SHY, between 4266.7 ± 57.7 ppm and 4566.7 ± 57.7 ppm for PAA, and between 3866.7 ± 57.7 and 4566.7 ± 57.7 ppm for BZK.

**Figure 2:**
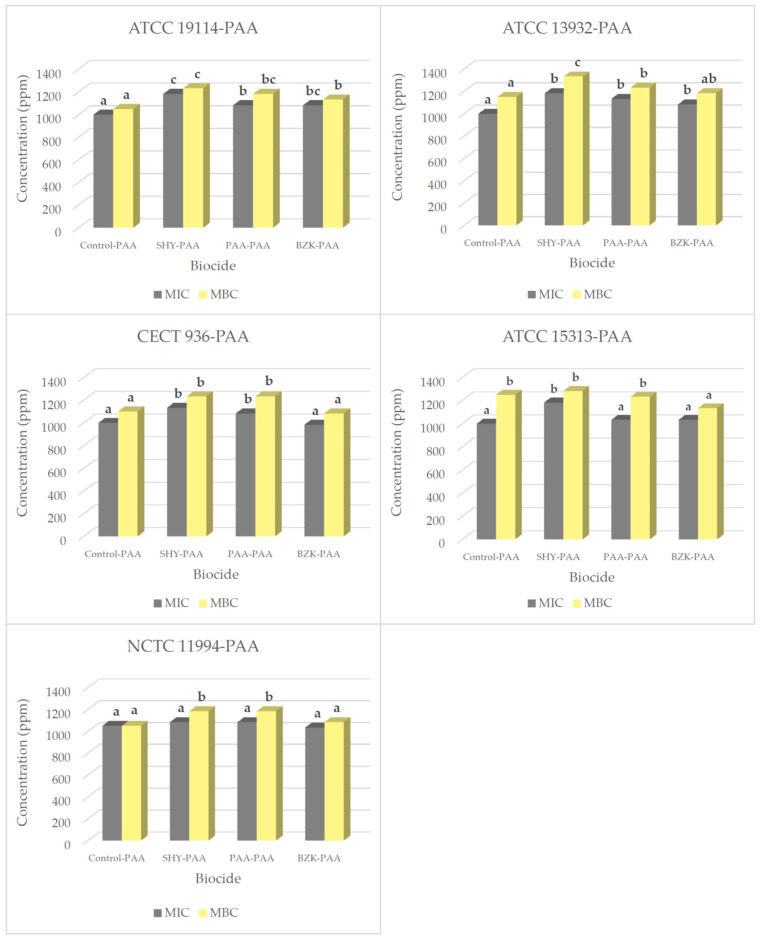
MICs and MBCs for peracetic acid (PAA) in several *L. monocytogenes* strains. These were either not adapted (control) or previously adapted to sodium hypochlorite (SHY), peracetic acid (PAA), or benzalkonium chloride (BZK). MIC and MBC values for peracetic acid (PAA) in strains not adapted to biocides (control–PAA), in strains adapted to sodium hypochlorite (SHY–PAA), in strains adapted to peracetic acid (PAA–PAA), and in strains adapted to benzalkonium chloride (BZK–PAA); *L. monocytogenes* ATCC 19114 serotype 4a; *L. monocytogenes* ATCC 13932 serotype 4b; *L. monocytogenes* CECT 936 serotype 1/2b; *L. monocytogenes* ATCC 15313 serotype 1/2a; *L. monocytogenes* NCTC 11994 serotype 4b. Data are the mean ± STD of three determinations. Data in the same graphics with no letters in common (superscript) are significantly different (*p* < 0.05).

Table 3 shows the results for the MIC and MBC of PAA in the strains adapted to the biocides. In most cases, the adapted strains showed higher MIC and MBC values for PAA than did the control strains. The MIC values for PAA in the *L. monocytogenes* strains adapted to SHY, PAA, and BZK lay in the range 983.3 ± 57.7 ppm to 1183.3 ± 28.9 ppm, in most cases differing from the control strains that had not been adapted to PAA (Figure 2).

The MBC values presented by the strains adapted to the various biocides when exposed to PAA after adaptation were disparate. An increase was observed in the strains of *L. monocytogenes* ATCC 19114, where values lay between 1133.3 ± 28.9 ppm and 1233.3 ± 57.7 ppm; ATCC 13932, which were between 1183.3 ± 28.9 ppm and 1333.3 ± 57.7 ppm; CECT 936, where there were higher values in the case of SHY–PAA and PAA–PAA at 1233.3 ± 57.7 ppm; and NCTC 11994, where values increased for SHY–PAA and PAA–PAA at 1183.3 ± 57.7 ppm. In contrast, MBC values showed a tendency to decrease relative to the control strains when there was exposure to PAA of the ATCC 15313 strain adapted to BZK, yielding values of 1133.3 ± 28.9 ppm.

**Figure 3:**
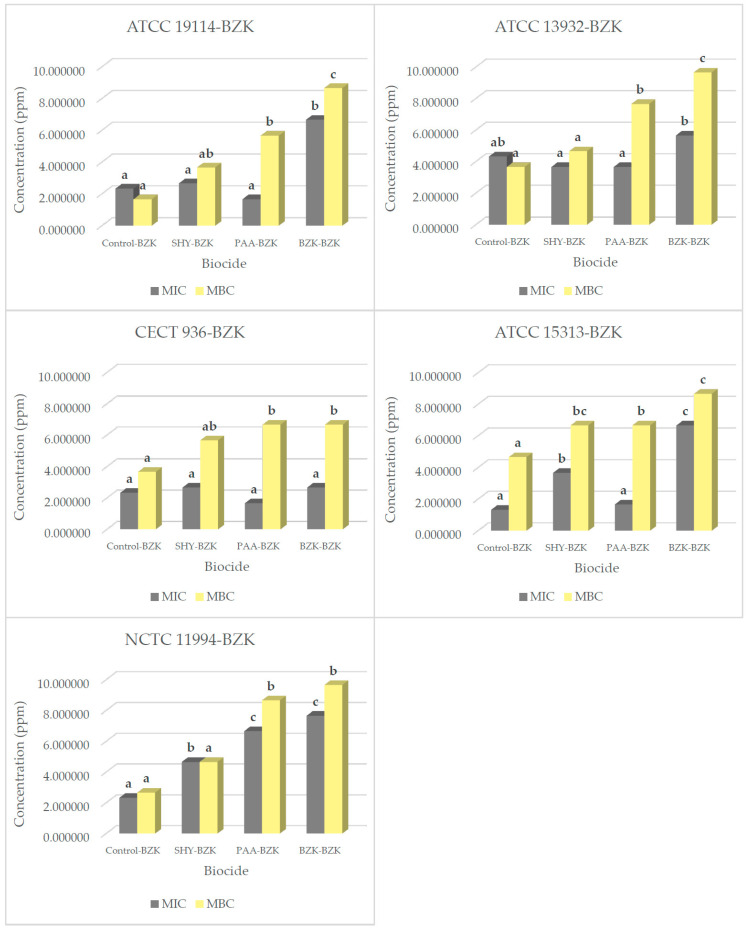
MICs and MBCs for benzalkonium chloride (BZK) in several *L. monocytogenes* strains. These were either not adapted (control) or previously adapted to sodium hypochlorite (SHY), peracetic acid (PAA), or benzalkonium chloride (BZK). MIC and MBC values for benzalkonium chloride (BZK) in strains not adapted to biocides (control–BZK), in strains adapted to sodium hypochlorite (SHY–BZK), in strains adapted to peracetic acid (PAA–BZK), and in strains adapted to benzalkonium chloride (BZK–BZK); *L. monocytogenes* ATCC 19114 serotype 4a; *L. monocytogenes* ATCC 13932 serotype 4b; *L. monocytogenes* CECT 936 serotype 1/2b; *L. monocytogenes* ATCC 15313 serotype 1/2a; *L. monocytogenes* NCTC 11994 serotype 4b. Data are the mean ± STD of three determinations. Data in the same graphic with no letters in common (superscript) are significantly different (*p* < 0.05).

Table 4 and Figure 3 show the data for the MIC and MBC of BZK in the strains adapted to biocides. Most strains exposed to increasing sub-inhibitory concentrations of the biocides saw a slight increase in their MIC for BZK. The MIC values for SHY–BZK and PAA–BZK were in the range between 1.7 ± 0.6 ppm and 4.7 ± 0.6 ppm, with the exception of the *L. monocytogenes* strain NCTC 11994 adapted to PAA, which presented a value of 6.7 ± 0.6 ppm. However, unlike the rest of those exposed, the strains adapted through exposure to BZK had an increased MIC in all cases, ranging between 2.7 ± 1.2 ppm and 7.7 ± 1.2 ppm, as may be seen in Figure 3.

The MBC values for all the strains analyzed showed an increase relative to those obtained for the control strains after adaptation to PAA or BZK, ranging between 5.7 ± 0.6 ppm and 9.7 ± 0.6 ppm, doubling in some cases, for example, the ATCC 19114 strain subjected to the test termed SHY–BZK, and being even more than double the value of the control strain in others, for instance, the ATCC 13932 strain with BZK–BZK. Hence, it may be stated that in general the treatment applied here to the set of strains led to a considerably heightened MBC value for biocides needed to affect them.

### 3.2. Antibiotic Resistance

Both before and after exposure to increasing sub-inhibitory concentrations of biocides, strains were examined for susceptibility to 30 antibiotics of clinical interest. The resistance of the strains to each of the antibiotics tested is shown in Table 5. Before exposure, the *L. monocytogenes* ATCC 19114 strain, of serotype 4a, showed resistance to 14 antibiotics; *L. monocytogenes* ATCC 13932, of serotype 4b, and *L. monocytogenes* ATCC 15313, of serotype 1/2a, to 15 in both instances; *L. monocytogenes* CECT 936, of serotype 1/2b, to 13; and *L. monocytogenes* NCTC 11994, of serotype 4b, to 17. Exposure of strains to increasing sub-inhibitory concentrations of the biocides was associated with reductions in susceptibility to several antibiotics, with some strains moving from the category of “susceptible”, which implies that infections due to the bacteria in question are likely to respond to a given antibiotic, to that of “resistant”, which denotes that infections caused by the bacteria concerned will probably not respond to the given antibiotic, in accordance with the guidelines used. In several instances, the increase in resistance was slight, with strains moving only from “susceptible” to “intermediate”, or from “intermediate” to “resistant”. For several cultures and various antibiotics, the diameter of the zones of inhibition in exposed cells decreased relative to unexposed cultures but did not exceed the resistance limit according to the EUCAST [25] and CLSI [26,27] guidelines.

## 4. Discussion

### 4.1. Determination of MIC and MBC Values for Biocides Before and After Adaptation of L. monocytogenes

The MIC data obtained for the biocides tested lay within the range of values previously observed when testing Gram-positive [19] and Gram-negative [17] bacteria. This confirms that they are effective antibacterial agents.

The disinfectant that required the highest concentrations to inhibit the growth of the *L. monocytogenes* strains studied was SHY. Previous studies [2,28,29] corroborate this fact in respect of *L. monocytogenes* strains of different serotypes. The lowest MICs were shown by BZK, a quaternary ammonium salt used as a biocide whose mechanism of bactericidal action consists of the dissociation of bacterial lipid bilayers, which gives rise to an increase in cell permeability with the consequent leakage of cellular contents.

In general terms, *L. monocytogenes* strains showed an acquired tolerance to biocides, especially BZK, after exposure to increasing sub-inhibitory concentrations of the substances under test. Studies recently carried out with Gram-positive bacteria [19] indicated that the increased and stable tolerance observed for some biocides, such as SHY, as a consequence of sublethal exposure of microorganisms could represent a risk of adaptation to antibacterial compounds. Improper use of biocides, for example, if they are inappropriately stored or are used in the presence of excessive amounts of organic matter, which are known to inactivate certain biocides, including chlorinated compounds, could represent a real threat of the development of reduced susceptibility to these chemicals [18].

It is important to highlight the exact difference between the terms “tolerance” and “resistance”. This is because on some occasions they have been used interchangeably, especially in relation to biocides [30]. Resistance should be understood as the insensitivity of a microorganism to a particular treatment under a specific set of conditions [31]. In contrast, the term “tolerance” is best applied to cases of strains in which the MIC of the antimicrobial increased compared with controls [32].

Most of the *L. monocytogenes* strains studied managed to adapt to the biocides tested. The strain that showed the greatest adaptation after exposure to increasing concentrations of SHY was *L. monocytogenes* ATCC 19114, requiring a concentration 2.0 times higher than the MIC. However, the biocide that showed the greatest levels of adaptation was BZK, with a need for a concentration 4.3 times higher than the MIC for *L. monocytogenes* strain NCTC 111994 and 5.2 times higher for ATCC 15313. Kimitsu et al. [33] have observed in various studies adaptation values five times higher than the MIC in Gram-positive bacteria such as methicillin-resistant *Staphylococcus aureus* (MRSA), which agrees with the results of the present research work. PAA was the biocide that presented the lowest requirement for increased concentrations as strains adapted, since this was 1.3 times higher for the *L. monocytogenes* strains ATCC 19114, ATCC 13932, and CETC 936.

This appears to be the first study in which *L. monocytogenes* strains were exposed to different biocides after adaptation so that cross-adaptation to the compounds could be verified. An increase in the MIC values was observed in most cases with respect to non-adapted strains, especially in those exposed to BZK. Other authors [34,35,36] have also found that adapted cells were less sensitive to a range of substances with completely different modes of action, as is the case with quaternary ammonium compounds and trisodium phosphate, chlorine, or phenol (carbolic acid). Alonso-Hernando et al. [37] also observed that a progressive increase in concentrations of different poultry decontaminants led to a lower susceptibility on the part of *L. monocytogenes* and *Salmonella enterica* strains, with MIC values that increased up to 2.7 times.

Similar to existing concerns about antibiotic resistance, concerns have been raised about the possibility of bacteria evading the effects of biocides, a fact that has been demonstrated by different authors [38,39,40,41,42,43,44,45,46,47,48,49,50,51]. Many types of pathogenic bacteria can be found in nature that have demonstrated the ability to adapt and develop tolerance to biocidal agents. He et al. [51] isolated bacterial strains from local community environments and found that 24% of isolates (268) collected from surfaces frequently disinfected with sprays or wipes containing BZK showed tolerance to this disinfectant (MIC > 3 ppm). This MIC value is in the same range as those of the present work for *L. monocytogenes*. Kampf [52] carried out a systematic search of scientific literature to evaluate the adaptive potential of bacteria when exposed to low doses of BZK. Most of the bacterial species considered (57) experienced an increase in their MIC values after exposure to the disinfectant. Among them, *Salmonella enterica* serotype Typhimurium showed the highest MIC for BZK (3000 ppm), followed by *Pseudomonas aeruginosa* (2500 ppm), *Enterobacter* spp. (1500 ppm), *Escherichia coli,* and *Staphylococcus saprophyticus* (1000 ppm). It should be noted that these values could exceed the in-use concentrations of BZK as a disinfectant agent.

Chlorine-tolerant bacteria are often detected in drinking water or drinking water distribution systems, with the most common being five bacterial genera: *Legionella*, *Sphingomonas*, *Mycobacterium*, *Bacillus*, and *Pseudomonas* [53]. In particular, it has been observed that *Sphingomonas* TS001, a chlorine-tolerant bacteria, showed only a 5% reduction in viability when exposed to 4 ppm sodium hypochlorite for 240 min [54]. Similar findings regarding increased alcohol tolerance have been reported in healthcare settings [55].

Consequently, the results reported here, as well as those from other authors, suggest that tolerance to some biocides could have an impact on any later use of other chemical compounds for cleaning and disinfection, significantly reducing their effectiveness.

### 4.2. Antibiotic Resistance

Infections caused by antibiotic-resistant bacteria are often difficult to treat, since many of the substances commonly used in clinical practice have to be ruled out as therapeutic options [17]. The widespread use of biocides and their consequent dissemination in the environment may contribute to adaptations in bacteria that lead to the development of a low level of susceptibility to antibacterial agents. Resistance mechanisms in bacteria are similar for both antimicrobials and biocides, so exposure to biocides can lead to cross-resistance to antibacterial agents [56].

The intent of the present study was to determine whether the adaptation of five strains of *L. monocytogenes* to three commonly used disinfectants brought about any change in antibiotic resistance with respect to strains not adapted to biocides. *L. monocytogenes* strains were tested against a panel of 30 antibiotics of clinical importance in both human and veterinary medicine before and after exposure to increasing sub-inhibitory concentrations of biocides. The antibiotics to which the *L. monocytogenes* strains were resistant are shown in Table 5.

All strains were susceptible to CN, P, TE, and KF before and after exposure to sub-inhibitory concentrations of biocides. These results coincide with what was stated by Granier et al. [57], who pointed out that most of the *L. monocytogenes* strains isolated from food between 1996 and 2006 were completely susceptible to penicillin and gentamycin but naturally resistant to cephalosporins. This is in accord with the outcomes of the current study, the sole exception being cephalothin (KF), a substance to which the strains showed susceptibility. Further, results relating to resistance to the antibiotics oxacillin (OX), ampicillin + sulbactam (SAM), enrofloxacin (ENR), ciprofloxacin (CIP), nalidixic acid (NA), clindamycin (DA), erythromycin (E), cefoxitin (FOX), cefotaxime (CTX), ceftriaxone (CRO), cefepime (FEP), rifampin (RD), nitrofurantoin (F), and aztreonam (ATM) were recorded.

Intrinsic resistance, one of the genetic bases of antibiotic resistance, refers to the innate insensitivity of bacteria to antimicrobial agents, which is determined by the biological characteristics of these bacteria, such as their structures and metabolic mechanisms. Gram-negative bacteria, for instance *Escherichia coli*, are generally not sensitive to neomycin antibiotics, while Gram-positive bacteria, such as *Streptococcus mutans* or *Staphylococcus aureus*, are less sensitive to antibiotics like streptomycin. Inherent resistance to antibiotics is mainly caused by the antibiotic resistance gene located on the chromosome of the bacteria, which is passed from generation to generation with specificity [58].

After exposure to increasing sub-inhibitory concentrations of SHY or BZK, a change in category of certain of the strains studied from susceptible to resistant was noted in respect of chloramphenicol, and a similar change from reduced susceptibility to resistance was seen for streptomycin. This coincides with the findings of Alonso-Hernando et al. [59], who observed an increase in the resistance of *L. monocytogenes* to streptomycin, cephalothin, and chloramphenicol after exposure to various decontaminating compounds. The results obtained by other authors, such as Capita et al. [60], are also in agreement with the above, since they pointed to a high prevalence of resistance in the case of cefoxitin, cefotaxime, cefepime, nalidixic acid, and oxacillin. The present research found that the strains were almost entirely resistant to the antibiotics mentioned (Table 3), both before and after exposure to sub-inhibitory concentrations of biocides. These results are of great concern, since some antibiotics from the aminoglycoside family, such as streptomycin, combined with penicillin, are the drugs of choice for the treatment of human listeriosis [61].

In many studies, a range of similar mechanisms have been found in resistance to biocides and to antibiotics. These include changes in cell envelopes, with a reduction in porins and modifications in lipopolysaccharides and other lipids, non-specific efflux pumps capable of expelling a wide range of compounds, overexpression of some genes, or alterations at the target site, as noted by the Scientific Committee on Emerging and Newly Identified Health Risks [62]. Some authors, such as Lavilla Lerma et al. [63], observed the existence of reduced susceptibility to antibiotics in strains of Gram-positive bacteria, in particular two species of *Enterococcus* spp., indicating that this might be due to non-specific resistance mechanisms such as efflux pumps. Similar findings were reported by Adkin et al. [64], who stated that sub-inhibitory concentrations of biocides can prime bacteria to become resistant to antibiotics, even in the absence of stable biocide tolerance. For *L. monocytogenes*, two MFS (Major Facilitator Superfamily) efflux pumps, MdrL and Lde, have been described as contributing to antibiotic resistance. Lde may confer resistance to hydrophilic fluoroquinolones. Furthermore, within *L. monocytogenes*, MdrL has been described as responsible for resistance to benzalkonium chloride when overexpressed [65]. Reducing the penetration of biocides into bacteria through modifications in cell envelopes or increasing the efflux of compounds through increased expression of efflux pumps are common mechanisms of cross-resistance between biocides and antibiotics [66].

However, the results noted here have revealed that, on occasion, some microorganisms can move from the category of “resistant” to “susceptible” or “with reduced susceptibility” after exposure to sub-inhibitory concentrations of biocides. Results of this sort were observed for antibiotics including kanamycin (K), ampicillin (AMP), amoxicillin (AML), ampicillin + sulbactam (SAM), ciprofloxacin (CIP), moxifloxacin (MXF), NA, nalidixic acid (NA), erythromycin (E), clarithromycin (CLR), meropenem (MEM), sulfamethoxazole-trimethoprim (SXT), chloramphenicol (C), rifampicin (RD), and nitrofurantoin (F).

Studies such as the work undertaken by Yong et al. [67] have highlighted that exogenous hydrogen sulfide (H_2_S) can produce modifications in resistance to the antibiotic gentamicin in microorganisms such as *Acinetobacter baumannii*, increasing their sensitivity to this antimicrobial substance. H_2_S triggered disturbances in redox homeostasis and cellular energy, which resulted in hypersensitivity to antibiotic attack. As a consequence, these authors propose that H_2_S might be used as an agent enhancing the action of antibiotics or reversing resistance in bacteria that do not produce this compound.

According to Allen et al. [68], a reversal in resistance, in both bacteria and other microorganisms, can occur through three mechanisms: (i) a resurgence of the sensitive ancestral genotype that prevailed before the evolution of resistance; (ii) the acquisition of additional alleles by the resistant lineage that decrease resistance without restoring the ancestral genotype; and (iii) the replacement of resistant genotypes by less resistant genotypes of the same species or strain that are not derived from the ancestral population. These types of reversal may respectively be termed isogenetic, paragenetic, and allogenetic reversal. Another noteworthy aspect of the reversal of antimicrobial resistance is changes in membrane permeability and the inhibition of efflux pumps [58]. Antibiotics can easily induce an overexpression of bacterial efflux pumps by forcing bacteria to expel more antibacterial drugs in an attempt to bring about a significant decrease in concentration at the target site [69]. Research continues into the existence of new adjuvants that can restore the effectiveness of existing antibiotics in a controlled manner [70].

## 5. Conclusions

This study confirms that exposure to sublethal concentrations of some biocides commonly used for cleaning and disinfecting surfaces and equipment in food industries, such as SHY, BZK, or PAA, can confer adaptation and cross-adaptation to other biocides in strains of *L. monocytogenes*. It is suggested that the adaptation of this bacteria to biocides could be caused, at least partially, by efflux pumps and changes in cell surface hydrophobicity. However, additional research is needed to confirm these findings. The mechanisms of adaptation to biocides must be investigated to develop strategies aimed at preventing or reducing this phenomenon, which may otherwise result in an increase in resistance to certain antibiotics, a worrying fact in the context of public health. In this context, data in the present work should be complemented with genotypic analysis. The hazards arising from exposure to sub-inhibitory concentrations of biocides must be considered when performing cleaning and disinfection operations in food processing environments.

## Figures and Tables

**Table 1:** MICs, MBCs, and maximum concentration of biocides allowing microbial growth after adaptation by *L. monocytogenes* strains. Data are expressed in ppm.

Disinfectant		Strain				
		ATCC 19114	ATCC 13932	CECT 936	ATCC 15313	NCTC 11994
SHY	MIC	3533.3 ± 28.9 ^a^_a_	3533.3 ± 28.9 ^a^_a_	3783.3 ± 28.9 ^b^_a_	3533.3 ± 28.9 ^a^_a_	3533.3 ± 28.9 ^a^_a_
	MBC	3883.3 ± 57.7 ^b^_b_	3683.3 ± 57.7 ^a^_b_	3883.3 ± 57.7 ^b^_b_	3683.3 ± 57.7 ^a^_b_	3983.3 ± 28.9 ^b^_b_
	Adaptation	6890.7 ± 1705.0 ^b^_c_	2770.3 ± 1100.2 ^a^_ab_	4594.3 ± 1136.8 ^b^_b_	4594.3 ± 1136.8 ^b^_c_	4594.3 ± 1136.8 ^b^_b_
PAA	MIC	1000.0 ± 25.0 ^a^_a_	1000.0 ± 25.0 ^a^_a_	1000.0 ± 25.0 ^a^_a_	1000.0 ± 25.0 ^a^_a_	1050.0 ± 25.0 ^a^_a_
	MBC	1050.0 ± 25.0 ^a^_a_	1150.0 ± 25.0 ^b^_b_	1100.0 ± 25.0 ^ab^_b_	1250.0 ± 25.0 ^c^_b_	1050.0 ± 25.0 ^a^_a_
	Adaptation	1312.7 ± 325.0 ^a^_b_	1312.7 ± 325.0 ^a^_b_	1312.7 ± 325.0 ^a^_b_	875.0 ± 216.5 ^a^_a_	1312.7 ± 325.0 ^a^_b_
BZK	MIC	2.3 ± 0.6 ^a^_a_	4.3 ± 0.6 ^b^_a_	2.3 ± 0.6 ^a^_a_	1.3 ± 0.6 ^a^_a_	2.3 ± 0.6 ^a^_a_
	MBC	1.7 ± 1.2 ^a^_a_	3.7 ± 1.2 ^a^_a_	3.7 ± 1.2 ^a^_ab_	4.7 ± 1.2 ^b^_b_	2.7 ± 1.2 ^a^_a_
	Adaptation	2.0 ± 0.5 ^a^_a_	9.9 ± 2.5 ^c^_b_	4.4 ± 1.1 ^b^_b_	6.7 ± 1.6 ^bc^_b_	9.9 ± 2.5 ^c^_b_

Data are the mean ± STD of three determinations. Data in the same row with no letters in common (superscript) are significantly different (*p* < 0.05). Data in the same column (values within each compound are compared) with no letters in common (subscript) are significantly different (*p* < 0.05). *L. monocytogenes* ATCC 19114 serotype 4a; *L. monocytogenes* ATCC 13932 serotype 4b; *L. monocytogenes* CECT 936 serotype 1/2b; *L. monocytogenes* ATCC 15313 serotype 1/2a; *L. monocytogenes* NCTC 11994 serotype 4b. SHY, sodium hypochlorite; PAA, peracetic acid; BZK, benzalkonium chloride. MIC, minimum inhibitory concentration; MBC, minimum bactericidal concentration; adaptation, maximum concentration of biocide that allowed microbial growth after several successive passages in culture medium with gradually increasing concentrations of the compound.

**Table 2:** MIC and MBC values for sodium hypochlorite (SHY) in *L. monocytogenes* strains previously adapted to various biocides. Data are expressed in ppm.

Adaptation	Strain									
	ATCC 19114		ATCC 13932		CECT 936		ATCC 15313		NCTC 11994	
	MIC	MBC	MIC	MBC	MIC	MBC	MIC	MBC	MIC	MBC
SHY-SHY	4666.7 ± 57.7 ^c^_b_	4666.7 ± 115.5 ^ab^_b_	4166.7 ± 57.7 ^a^_a_	4566.7 ± 115.5 ^ab^_a_	4466.7 ± 57.7 ^b^_c_	4466.7 ± 115.5 ^a^_b_	4466.7 ± 57.7 ^b^_b_	4666.7 ± 115.5 ^ab^_b_	4666.7 ± 57.7 ^c^_b_	4766.7 ± 115.5 ^b^_c_
PAA-SHY	4366.7 ± 57.7 ^c^_a_	4566.7 ± 57.7 ^b^_ab_	4066.7 ± 57.7 ^b^_a_	4566.7 ± 57.7 ^b^_a_	4066.7 ± 57.7 ^b^_b_	4466.7 ± 57.7 ^b^_b_	4266.7 ± 57.7 ^c^_a_	4566.7 ± 57.7 ^b^_b_	3766.7 ± 57.7 ^a^_a_	4266.7 ± 57.7 ^a^_b_
BZK-SHY	4366.7 ± 115.5 ^c^_a_	4466.7 ± 57.7 ^c^_a_	4066.7 ± 115.5 ^b^_a_	4566.7 ± 57.7 ^c^_a_	3466.7 ± 115.5 ^a^_a_	3866.7 ± 57.7 ^a^_a_	4066.7 ± 115.5 ^b^_a_	4466.7 ± 57.7 ^c^_a_	3666.7 ± 115.5 ^a^_a_	4066.7 ± 57.7 ^b^_a_

Data are the mean ± STD of three determinations. Data in the same row with no letters in common (superscript; MIC and MBC values were compared separately) are significantly different (*p* < 0.05). Data in the same row with significant differences observed between MIC and MBC for the same strain are underlined. Data in the same column with no letters in common (subscript) are significantly different (*p* < 0.05). MIC and MBC values for SHY in the SHY-adapted strain (SHY–SHY); for SHY in the PAA-adapted strain (PAA–SHY); and for SHY in the BZK-adapted strain (BZK–SHY). For additional interpretation, see Table 1.

**Table 3:** MIC and MBC values for peracetic acid (PAA) in *L. monocytogenes* strains previously adapted to various biocides. Data are expressed in ppm.

Adaptation	Strain									
	ATCC 19114		ATCC 13932		CECT 936		ATCC 15313		NCTC 11994	
	MIC	MBC	MIC	MBC	MIC	MBC	MIC	MBC	MIC	MBC
SHY–PAA	1183.3 ± 28.9 ^a^_b_	1233.3 ± 57.7 ^ab^_a_	1183.3 ± 28.9 ^a^_a_	1333.3 ± 57.7 ^b^_b_	1133.3 ± 28.9 ^a^_b_	1233.3 ± 57.7 ^ab^_b_	1183.3 ± 28.9 ^a^_b_	1283.3 ± 57.7 ^ab^_b_	1083.3 ± 28.9 ^a^_a_	1183.3 ± 57.7 ^a^_b_
PAA–PAA	1083.3 ± 28.9 ^a^_a_	1183.3 ± 28.9 ^a^_ab_	1133.3 ± 28.9 ^a^_a_	1233.3 ± 28.9 ^a^_a_	1083.3 ± 28.9 ^a^_ab_	1233.3 ± 28.9 ^a^_b_	1033.3 ± 28.9 ^a^_a_	1233.3 ± 28.9 ^a^_b_	1083.3 ± 28.9 ^a^_a_	1183.3 ± 28.9 ^a^_b_
BZK–PAA	1083.3 ± 57.7 ^a^_ab_	1133.3 ± 28.9 ^a^_b_	1083.3 ± 57.7 ^a^_a_	1183.3 ± 28.9 ^a^_a_	983.3 ± 57.7 ^a^_a_	1083.3 ± 28.9 ^a^_a_	1033.3 ± 57.7 ^a^_a_	1133.3 ± 28.9 ^a^_a_	1033.3 ± 57.7 ^a^_a_	1083.3 ± 28.9 ^a^_a_

Data are the mean ± STD of three determinations. Data in the same row with no letters in common (superscript; MIC and MBC values were compared separately) are significantly different (*p* < 0.05). Data in the same row with significant differences observed between MIC and MBC for the same strain are underlined. Data in the same column with no letters in common (subscript) are signifi-cantly different (*p* < 0.05). MIC and MBC values for PAA in the SHY-adapted strain (SHY–PAA); for PAA in the PAA-adapted strain (PAA–PAA); and for PAA in the BZK-adapted strain (BZK–PAA). For additional interpretation, see Table 1.

**Table 4:** MIC and MBC values for benzalkonium chloride (BZK) in *L. monocytogenes* strains previously adapted to various biocides. Data are expressed in ppm.

Adaptation	Strain									
	ATCC 19114		ATCC 13932		CECT 936		ATCC 15313		NCTC 11994	
	MIC	MBC	MIC	MBC	MIC	MBC	MIC	MBC	MIC	MBC
SHY–BZK	2.7 ± 0.6 ^a^_b_	3.7 ± 1.2 ^a^_a_	3.7 ± 0.6 ^a^_a_	4.7 ± 1.2 ^a^_a_	2.7 ± 0.6 ^a^_a_	5.7 ± 1.2 ^a^_a_	3.7 ± 0.6 ^a^_b_	6.7 ± 1.2 ^b^_ab_	4.7 ± 0.6 ^b^_a_	4.7 ± 1.2 ^a^_a_
PAA–BZK	1.7 ± 0.6 ^a^_a_	5.7 ± 0.6 ^a^_a_	3.7 ± 0.6 ^b^_a_	7.7 ± 0.6 ^b^_b_	1.7 ± 0.6 ^a^_a_	6.7 ± 0.6 ^ab^_a_	1.7 ± 0.6 ^a^_a_	6.7 ± 0.6 ^a b^_a_	6.7 ± 0.6 ^c^_b_	8.7 ± 0.6 ^c^_b_
BZK–BZK	6.7 ± 1.2 ^b^_c_	8.7 ± 0.6 ^b^_b_	5.7 ± 1.2 ^b^_b_	9.7 ± 0.6 ^b^_c_	2.7 ± 1.2 ^a^_a_	6.7 ± 0.6 ^a^_a_	6.7 ± 1.2 ^b^_c_	8.7 ± 0.6 ^b^_b_	7.7 ± 1.2 ^b^_b_	9.7 ± 0.6 ^b^_b_

Data are the mean ± STD of three determinations. Data in the same row with no letters in common (superscript; MIC and MBC values were compared separately) are significantly different (*p* < 0.05). Data in the same row with significant differences observed between MIC and MBC for the same strain are underlined. Data in the same column with no letters in common (subscript) are significantly different (*p* < 0.05). MIC and MBC values for BZK in the SHY-adapted strain (SHY–BZK); for BZK in the PAA-adapted strain (PAA–BZK); and for BZK in the BZK-adapted strain (BZK–BZK). For additional interpretation, see Table 1.

**Table 5:** Antibiotic resistance in various strains of *L. monocytogenes*.

*L. monocytogenes* Strains	Antibiotics																													
	S	CN	AK	K	VA	AMP	P	OX	AML	SAM	ENR	CIP	MXF	NA	TE	DA	E	CLR	IMP	MEM	KF	FOX	CTX	CRO	FEP	SXT	C	RD	F	ATM
4a ^1^ non exposed	I	S	S	I	I	S	S	R	I	R	R	R	S	R	S	R	R	I	S	I	S	R	R	R	R	S	S	R	R	R
4a exposed to SHY	R	S	I	R	R	S	S	R	R	R	R	R	R	R	S	R	S	R	S	I	S	R	R	R	R	R	R	R	R	R
4a exposed to PAA	I	S	S	I	I	S	S	R	I	R	R	R	R	R	S	R	S	I	S	I	S	R	R	R	R	R	S	R	R	R
4a exposed to BZK	I	S	S	I	I	S	S	R	I	I	R	S	S	R	S	R	S	I	S	I	S	R	R	R	R	S	S	R	R	R
4b1 ^2^ non exposed	I	S	S	I	I	S	S	R	R	R	R	R	R	R	S	R	S	I	S	I	S	R	R	R	R	S	S	R	R	R
4b1 exposed to SHY	I	S	S	I	I	R	S	R	R	R	R	R	S	R	S	R	S	I	R	S	S	R	R	R	R	S	S	R	S	R
4b1 exposed to PAA	I	S	S	I	I	S	S	R	I	I	R	R	S	R	S	R	S	I	S	I	S	R	R	R	R	R	S	R	R	R
4b1 exposed to BZK	I	S	S	I	I	S	S	R	I	R	R	S	S	R	S	R	S	I	S	S	S	R	R	R	R	S	S	R	R	R
1/2b ^3^ non exposed	I	S	S	R	I	S	S	R	R	R	R	S	S	R	S	R	S	S	S	S	S	R	R	R	R	R	S	I	S	R
1/2b exposed to SHY	I	S	S	I	I	S	S	R	I	R	R	R	S	R	S	R	S	I	S	S	S	R	R	R	R	S	S	R	R	R
1/2b exposed to PAA	I	S	S	I	S	S	S	R	I	I	R	R	S	R	S	R	S	I	S	S	S	R	R	R	R	R	S	I	R	R
1/2b exposed to BZK	I	S	S	I	I	S	S	R	I	R	R	R	S	R	S	R	S	I	S	S	S	R	R	R	R	S	S	R	I	R
1/2a ^4^ non exposed	S	S	S	R	S	S	S	R	S	I	R	R	R	R	S	R	S	I	S	S	S	R	R	R	R	R	S	R	R	R
1/2a exposed to SHY	I	S	S	I	I	S	S	R	I	R	R	R	R	R	S	R	S	I	S	I	S	R	R	R	R	S	S	R	R	R
1/2a exposed to PAA	I	S	S	I	R	S	S	R	I	I	R	R	R	R	S	R	S	S	S	S	S	R	R	R	R	S	S	R	R	R
1/2a exposed to SHY	S	S	S	S	S	S	S	R	I	I	R	R	S	R	S	R	S	I	S	I	S	R	R	R	R	R	S	R	R	R
4b2 ^5^ non exposed	I	S	S	I	R	S	S	R	I	R	R	R	R	R	S	R	R	R	S	I	S	R	R	R	R	R	S	R	R	I
4b2 exposed to SHY	I	S	S	I	S	S	S	R	I	I	R	S	R	S	S	R	S	S	S	S	S	R	R	R	R	S	S	S	S	R
4b2 exposed to PAA	R	S	S	I	I	S	S	R	I	I	R	R	R	R	S	R	R	S	S	I	S	R	R	R	R	R	S	R	R	R
4b2 exposed to BZK	I	S	S	I	I	S	S	R	I	I	R	S	S	R	S	R	R	I	S	I	S	R	R	R	R	S	S	R	R	R

^1^ ATCC 19114 (serotype 4a), ^2^ ATCC 13932 (serotype 4b), ^3^ CETC 936 (serotype 1/2b), ^4^ ATCC 15313 (serotype 1/2a), ^5^ NCTC 11884 (serotype 4b). R, resistant strain; I, intermediate strain (with reduced susceptibility); S, susceptible strain. Exposed strains with increased resistance to antibiotics relative to unexposed strains are shaded in orange; exposed strains with decreased resistance to antibiotics relative to unexposed strains are shaded in green. An increase in resistance was defined as a change from S, before exposure, to R, after exposure; from S, before exposure, to I, after exposure; or from I, before exposure, to R, after exposure, in accordance with the EUCAST and CLSI guidelines. S (streptomycin, 10 µg), CN (gentamycin, 10 µg), AK (amikacin, 30 µg), K (kanamycin, 30 µg), VA (vancomycin, 30 µg), AMP (ampicillin, 10 µg), P (penicillin, 10 units), OX (oxacillin, 1 µg), AML (amoxicillin, 10 µg), SAM (ampicillin plus sulbactam, 20 µg), ENR (enrofloxacin, 5 µg), CIP (ciprofloxacin, 5 µg), MXF (moxifloxacin, 5 µg), NA (nalidixic acid, 30 µg), TE (tetracycline, 30 µg), DA (clindamycin, 2 µg), E (erythromycin, 15 µg), CLR (clarithromycin, 15 µg), IMP (imipenem, 10 µg), MEM (meropenem, 10 µg), KF (cephalothin, 30 µg), FOX (cefoxitin, 30 µg), CTX (cefotaxime, 30 µg), CRO (ceftriaxone, 30 µg), FEP (cefepime, 30 µg), SXT (sulfamethoxazole-trimethoprim, 25 µg), C (chloramphenicol, 30 µg), RD (rifampin, 5 µg), F (nitrofurantoin, 300 µg) and ATM (aztreonam, 30 µg).

## Data Availability

The data presented in this study are available on request.
